# Supplementary material for: Characterizing immune variation and diagnostic indicators of preeclampsia by single-cell RNA sequencing and machine learning
Source: Commun Biol. 2024 Jan 5;7:32. doi: 10.1038/s42003-023-05669-2 (PMC10770323; doi:10.1038/s42003-023-05669-2)
Supplement: Supplementary file 2 — Supplementary Information [file 42003_2023_5669_MOESM2_ESM.pdf]

Characterizing immune variation and diagnostic indicators of preeclampsia by single-cell RNA sequencing and machine learning

Wenwen Zhou, Yixuan Chen, Yuhui Zheng, Yong Bai, Jianhua Yin, Xiao-Xia Wu, Mei Hong, Langchao Liang, Jing Zhang, Ya Gao, Ning Sun, Jiankang Li, Yiwei Zhang, Linlin Wu, Xin Jin, Jianmin Niu

Supplementary Figure 1. Cellular composition of normal pregnancy and preeclampsia, related to Figure 1

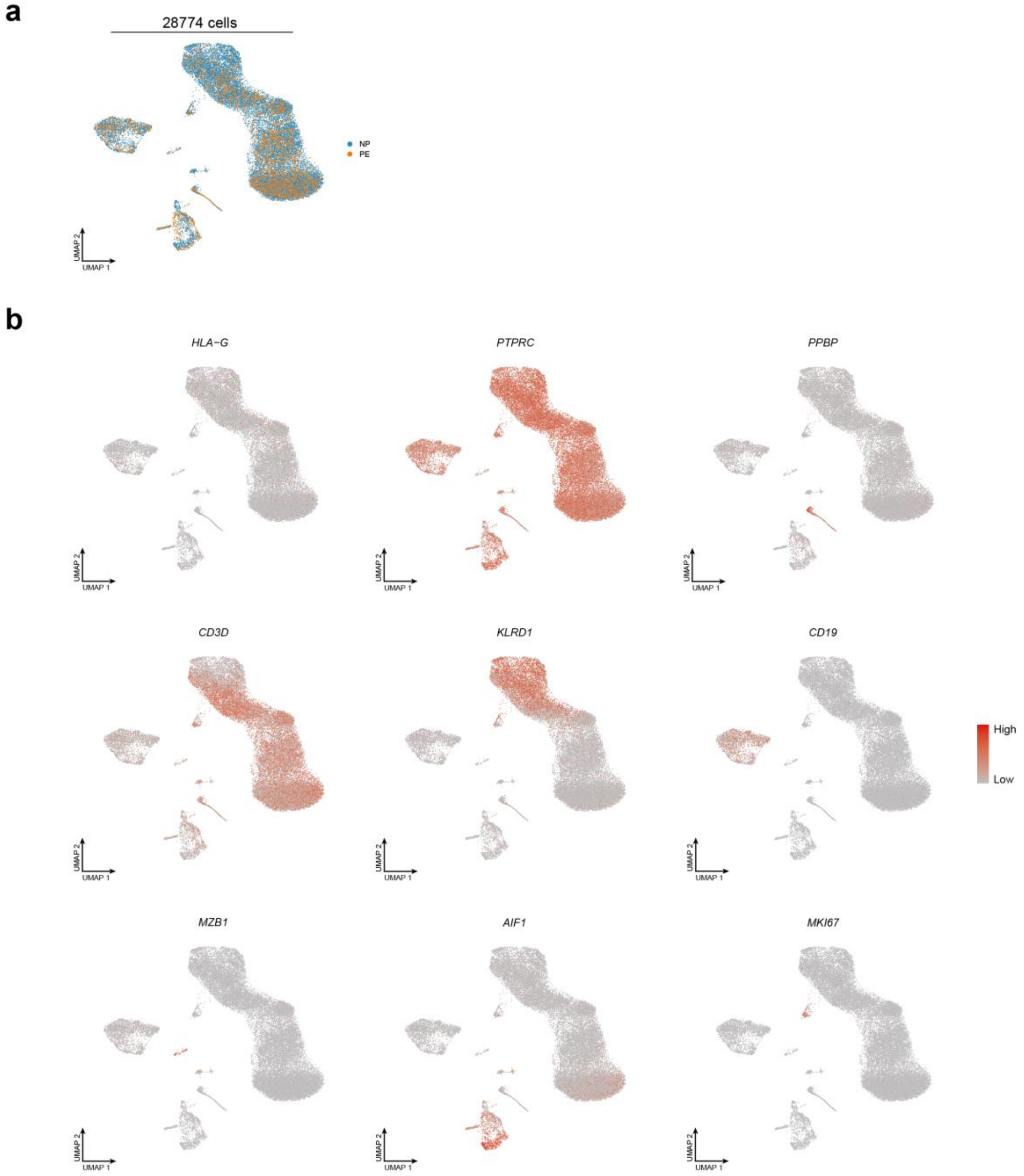

(a) UMAP plot shows the 28,774 PBMCs obtained from NP and PE.

(b) UMAP plots show the expression of canonical markers in NP and PE. The distribution of *HLA-G* shows no trophoblast cell cluster was identified, and all cells investigated in this study were CD45$^+$ (*PTPRC*) immune cells.

Supplementary Figure 2. Composition and functional analysis of B cell subsets, related to Figure 2

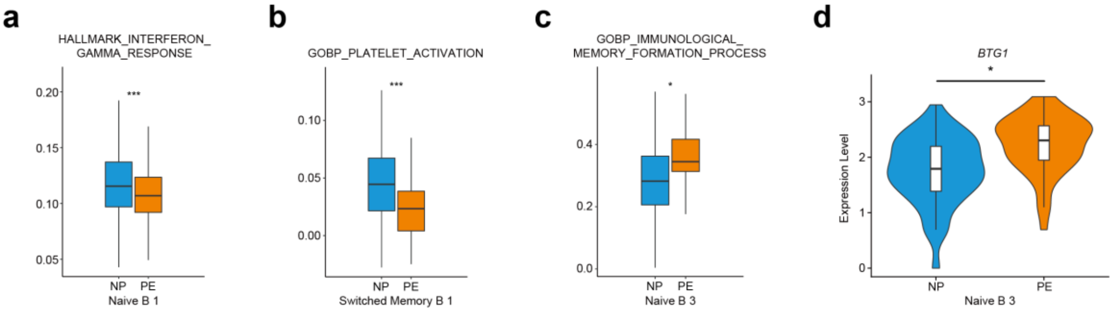

(a-c) Box plot shows the expression of functional pathways in switched memory B 1 cell, naïve B 1 cell, and naïve B 3 cell respectively. Student's t-test. *adjust $P < 0.05$, ***adjust $P < 0.001$.

(d) Violin plot shows the expression of *BTG1* in naïve B 3 cell. Wilcoxon rank-sum test. *adjust $P < 0.05$.

Supplementary Figure 3. Functional analysis of T and NK cell subsets, related to Figure 3

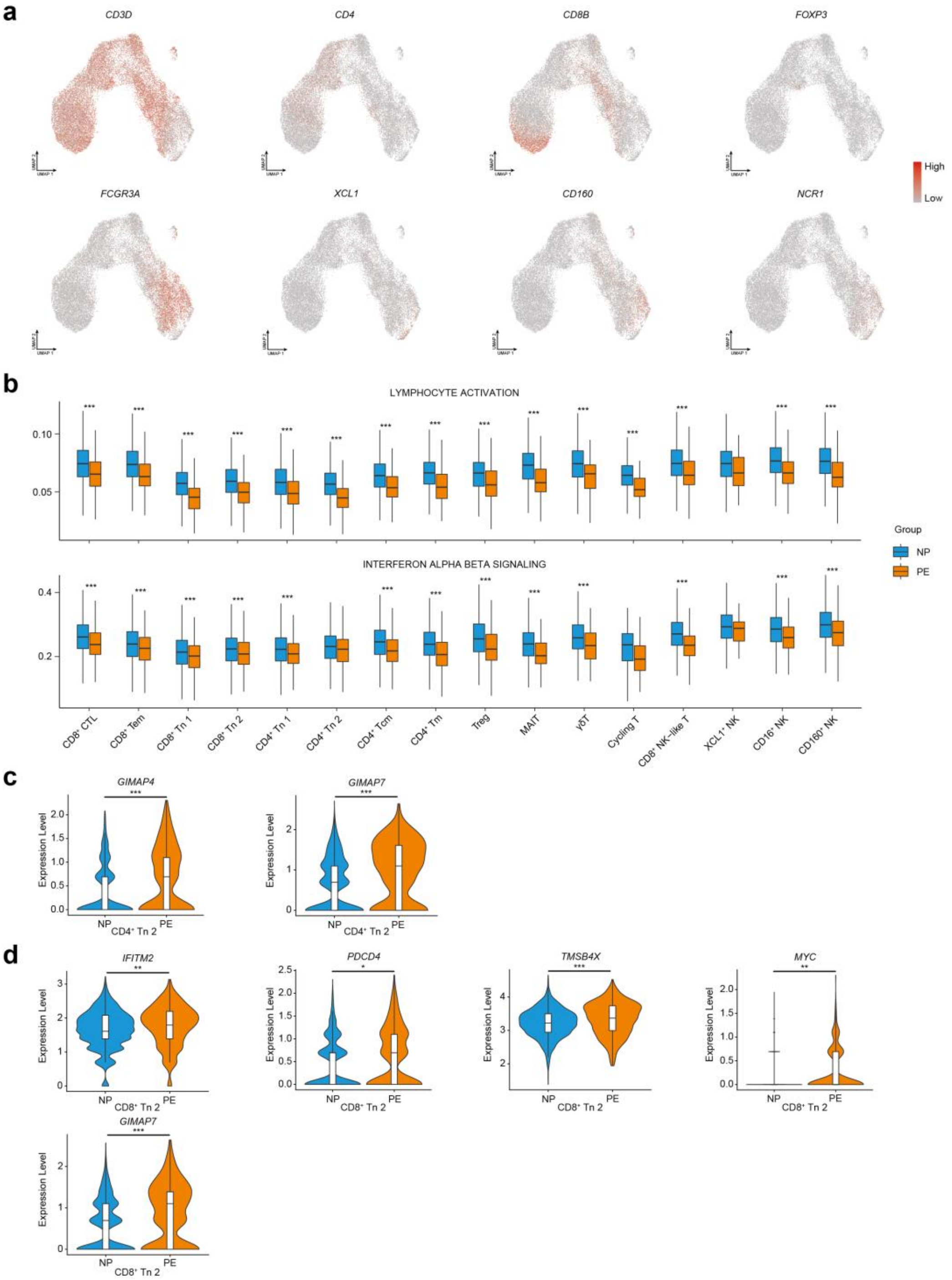

(a) UMAP plots show the expression of canonical markers of T and NK cell subsets in NP and PE.

(b) Box plots show the reduced expression of functional pathways in total T and NK cell subsets. Student's t-test. ***adjust $P < 0.001$.

(c) Violin plots show the expression of functional genes in $CD4^+$ Tn 2 cell. Wilcoxon rank-sum test. ***adjust $P < 0.001$.

(d) Violin plots show the expression of functional genes in $CD8^+$ Tn 2 cell, Wilcoxon rank-sum test. *adjust $P < 0.05$, **adjust $P < 0.01$, ***adjust $P < 0.001$.

Supplementary Figure 4. Functional analysis of myeloid cell subsets, related to Figure 4

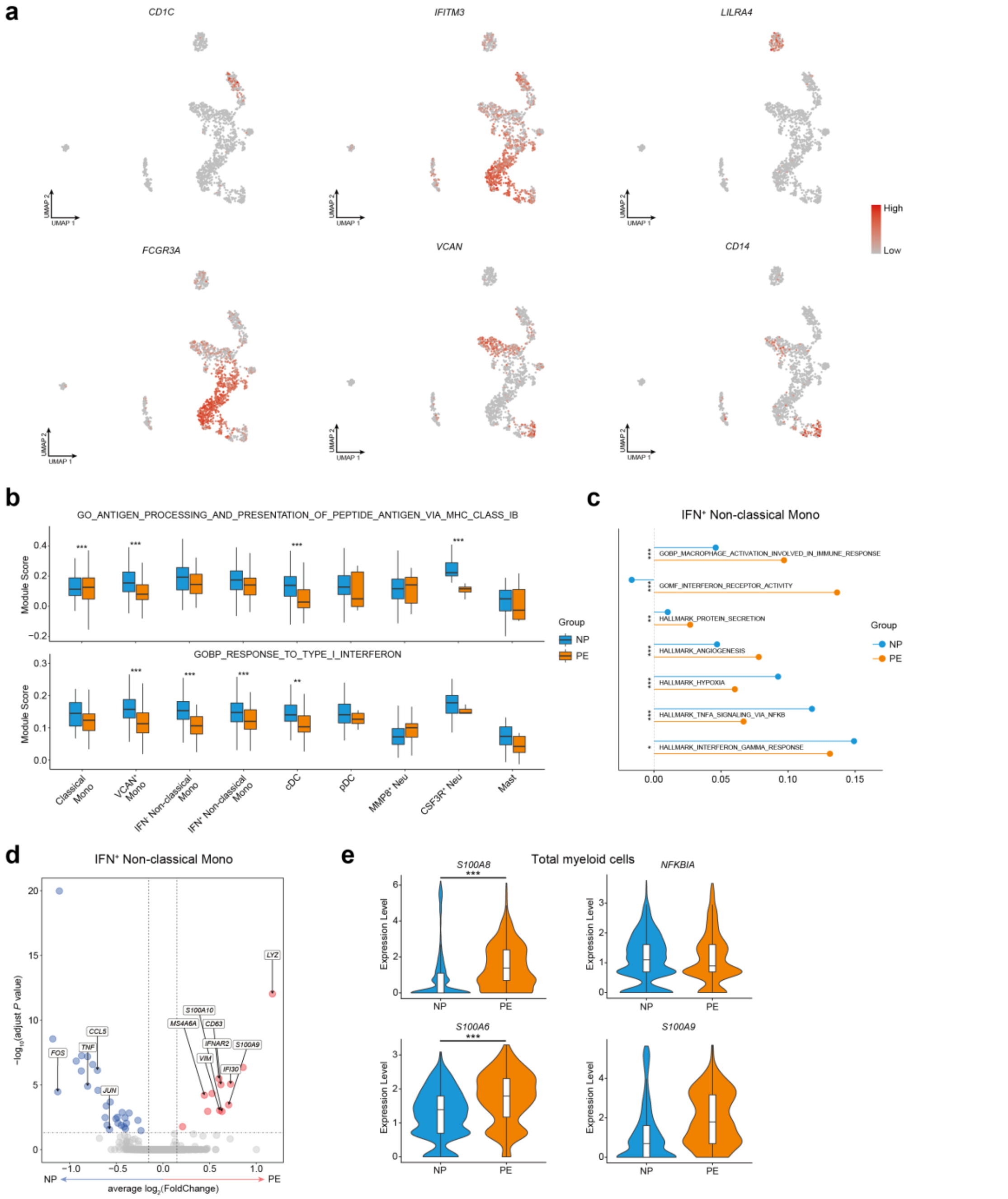

(a) UMAP plots show the expression of canonical markers of myeloid cell subsets in NP and PE.

(b) Box plots show the reduced expression of MHC-I-related pathway in all myeloid cell subsets. Student's t-test. ***adjust $P < 0.001$.

(c) Line and dot plot shows the expression of functional pathways in IFN^{+} non-classical Mono. Student's t-test. *adjust $P < 0.05$, **adjust $P < 0.01$, ***adjust $P < 0.001$.

(d) Volcanic plot shows the down-regulated DEGs (blue dots) and up-regulated DEGs (red dots) between NP and PE in IFN^{+} non-classical Mono. Wilcoxon rank-sum test.

(e) Violin plots show the expression of functional genes in total myeloid cells, Wilcoxon rank-sum test. ***adjust $P < 0.001$

Supplementary Figure 5. Additional evaluation metrics showing the performance of the four cell-type-specific models for preeclampsia diagnosis, related to Figure 6

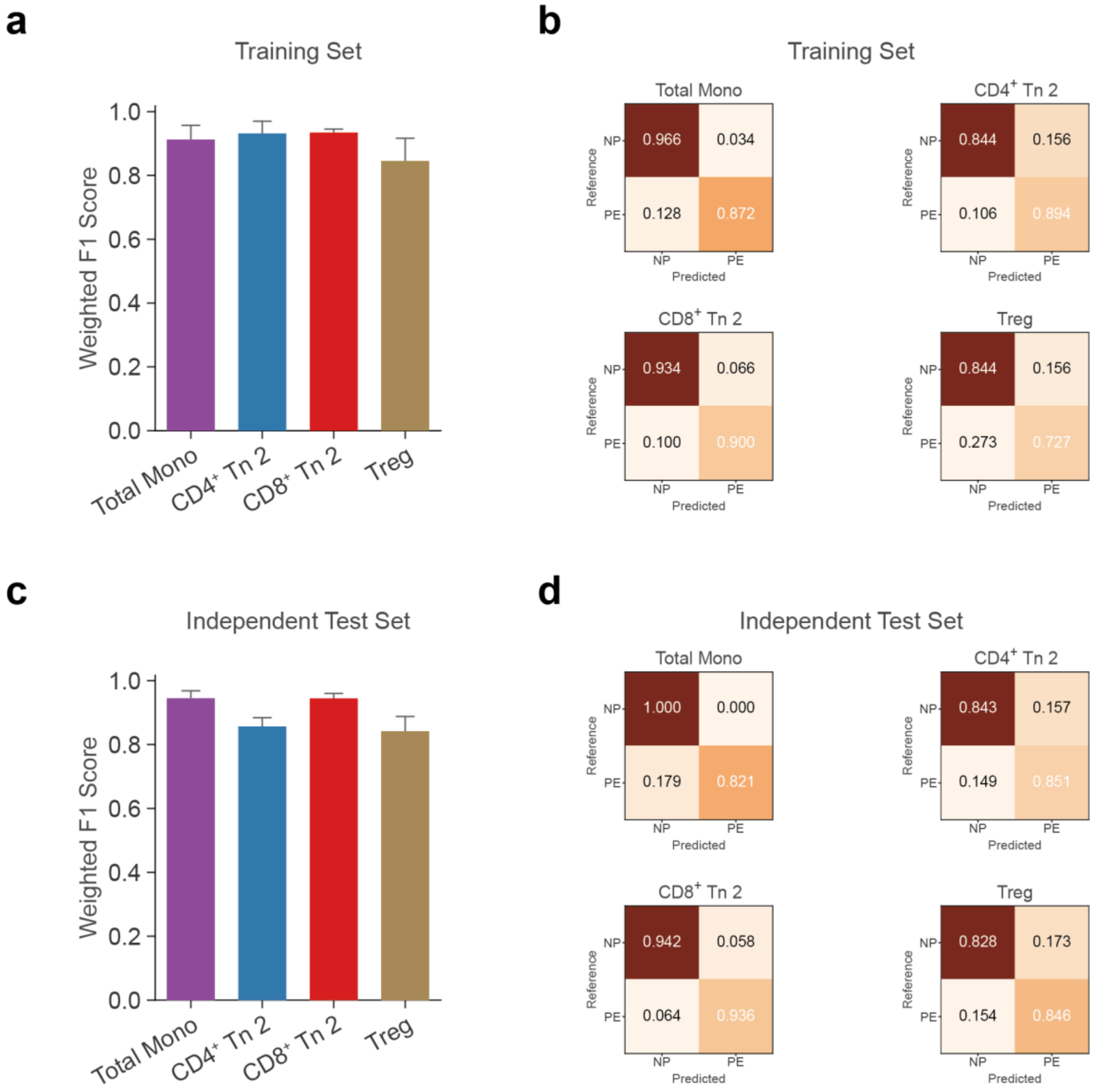

(a-b) The weighted F1 scores (a) and confusion matrix results (b) evaluated using the training sets.

(c-d) The weighted F1 scores (c) and confusion matrix results (d) evaluated using the independent test sets.

Supplementary Table 1. The number of single cells and pseudo-cells in the training dataset and the independent test dataset for each cell type after data preprocessing. The number of selected genes by feature selection is also shown. Positives and Negatives: the number of pseudo-cells generated using the single cells collected from PE patients and NP controls, respectively.

Cell type	Single cells	Training set		Independent test set		Select genes
		Single cells	Pseudo-cells (Positives, Negatives)	Single cells	Pseudo-cells (Positive, Negative)	
Total Mono	870	608	126 (39, 87)	262	55 (17, 38)	28
$CD4^+$ Tn 2	1571	1097	226 (47, 179)	474	98 (20, 78)	19
$CD8^+$ Tn 2	2477	1731	354 (80, 274)	746	152 (34, 118)	35
Treg	837	590	123 (33, 90)	247	54 (14, 40)	8

Supplementary Table 2. Optimal hyperparameter values determined by the 5-fold cross validation approach using the training set for each cell type-specific random forest (RF)-based classifier. Parameter descriptions for the RF model can be found on scikit-learn website. Default values are used for parameters otherwise.

Parameter	Search space	Cell type-specific model	Optimal value
n_estimators	Discrete values in [20,200], step=19	Total Mono	200
		$CD4^+$ Tn 2	200
		$CD8^+$ Tn 2	52
		Treg	126
min_samples_split	Discrete values in {2, 5, 10}	Total Mono	10
		$CD4^+$ Tn 2	10
		$CD8^+$ Tn 2	5
		Treg	10
min_samples_leaf	Discrete values in {1, 2, 4}	Total Mono	2
		$CD4^+$ Tn 2	2
		$CD8^+$ Tn 2	2
		Treg	4
max_depth	Discrete values in [2,20], step=1	Total Mono	9
		$CD4^+$ Tn 2	9
		$CD8^+$ Tn 2	14
		Treg	11
