# Supplementary material for: Characterizing immune variation and diagnostic indicators of preeclampsia by single-cell RNA sequencing and machine learning
Source: Commun Biol. 2024 Jan 5;7:32. doi: 10.1038/s42003-023-05669-2 (PMC10770323; doi:10.1038/s42003-023-05669-2)
Supplement: Supplementary file 3 — Description of Additional Supplementary Files [file 42003_2023_5669_MOESM3_ESM.docx]

Description of Additional Supplementary Files

**File name:** Supplementary Data 1

**Description:** Detailed clinical information of each sample in PE and NP, related to Figure 1.

**File name:** Supplementary Data 2

**Description:** Top 20 differentially expressed genes of major cell types, related to Figure 1.

**File name:** Supplementary Data 3

**Description:** The source data behind the graphs in the paper
