# Supplementary material for: Characterizing immune variation and diagnostic indicators of preeclampsia by single-cell RNA sequencing and machine learning
Source: Commun Biol. 2024 Jan 5;7:32. doi: 10.1038/s42003-023-05669-2 (PMC10770323; doi:10.1038/s42003-023-05669-2)
Supplement: Supplementary file 7 — Reporting Summary [file 42003_2023_5669_MOESM7_ESM.pdf]

nature portfolio

Corresponding author(s): Jianmin Niu, Xin Jin, Linlin Wu

Last updated by author(s): Nov 27, 2023

Reporting Summary

Nature Portfolio wishes to improve the reproducibility of the work that we publish. This form provides structure for consistency and transparency in reporting. For further information on Nature Portfolio policies, see our Editorial Policies and the Editorial Policy Checklist.

Statistics

For all statistical analyses, confirm that the following items are present in the figure legend, table legend, main text, or Methods section.

n/a	Confirmed	
☐	☒	The exact sample size (*n*) for each experimental group/condition, given as a discrete number and unit of measurement
☐	☒	A statement on whether measurements were taken from distinct samples or whether the same sample was measured repeatedly
☐	☒	The statistical test(s) used AND whether they are one- or two-sided<br>*Only common tests should be described solely by name; describe more complex techniques in the Methods section.*
☒	☐	A description of all covariates tested
☐	☒	A description of any assumptions or corrections, such as tests of normality and adjustment for multiple comparisons
☐	☒	A full description of the statistical parameters including central tendency (e.g. means) or other basic estimates (e.g. regression coefficient) AND variation (e.g. standard deviation) or associated estimates of uncertainty (e.g. confidence intervals)
☐	☒	For null hypothesis testing, the test statistic (e.g. *F*, *t*, *r*) with confidence intervals, effect sizes, degrees of freedom and *P* value noted<br>*Give P values as exact values whenever suitable.*
☒	☐	For Bayesian analysis, information on the choice of priors and Markov chain Monte Carlo settings
☒	☐	For hierarchical and complex designs, identification of the appropriate level for tests and full reporting of outcomes
☒	☐	Estimates of effect sizes (e.g. Cohen's *d*, Pearson's *r*), indicating how they were calculated

Our web collection on statistics for biologists contains articles on many of the points above.

Software and code

Policy information about availability of computer code

Data collection	DNBelab C4 system (BGl, v1.0) was used to process scRNA-seq data and generate single-cell expression matrix.
Data analysis	Seurat object was created by the Seurat R package(v4.3.0), with R(v4.1.1). Harmony R package(v0.1.0) was used for batch correction. ClusterProfiler R package(v4.2.2) was used to perform functional analysis of significantly up-and down-regulated genes respectively. We used CellPhoneDB (v3.1.0) to analyze cell-cell interactions between immune cells."Velocyto"(v0.17.16) and "scVelo"Python package (v0.2.4) were used for RNA velocity estimation. The mutual information(MI)-based algorithm, the Boruta algorithm, scikit-learn(v1.0.2), the SHAP method (version 0.39.0) were used for machine learning.

For manuscripts utilizing custom algorithms or software that are central to the research but not yet described in published literature, software must be made available to editors and reviewers. We strongly encourage code deposition in a community repository (e.g. GitHub). See the Nature Portfolio guidelines for submitting code & software for further information.

Data

Policy information about availability of data

All manuscripts must include a data availability statement. This statement should provide the following information, where applicable:

- Accession codes, unique identifiers, or web links for publicly available datasets
- A description of any restrictions on data availability
- For clinical datasets or third party data, please ensure that the statement adheres to our policy

The normalized gene expression matrix of scRNA-seq data was deposited in the CNGB Nucleotide Sequence Archive (CNSA) (accession code: CNP0003201) of the China National GeneBank DataBase (CNGBdb). Other relevant data are available from the corresponding author upon reasonable request.

Research involving human participants, their data, or biological material

Policy information about studies with human participants or human data. See also policy information about sex, gender (identity/presentation), and sexual orientation and race, ethnicity and racism.

Reporting on sex and gender	As this study focuses on pregnancy complication, both the disease and control groups consist of pregnant women. To eliminate the potential influence of fetal gender on immune responses of participants, the fetal gender ratio in both groups was about 1:1, and thus this study does not include sex-and gender-based analysis.
Reporting on race, ethnicity, or other socially relevant groupings	As this study is a case/control study on pregnancy complication, all subjects were Asian pregnant women, the grouping in this article was based on diagnostic guidelines and clinical diagnosis. Race, ethnicity and other socially relevant factors were not included in grouping.
Population characteristics	Preeclampsia were diagnosed according to the Guidelines for the Diagnosis and Treatment of hypertensive Disorders in Pregnancy (2020) of the Chinese Society of Obstetrics and Gynecology, Chinese Medical Association. One-to-two propensity score matching (PSM) was performed to match uncomplicated cohort participants as controls based on maternal age and gestational age of blood sampling. Some of the hemolysis samples were eliminated. Finally,a total of 8 patients with preeclampsia and 15 normal pregnant women were included in this study, all of whom were women aged from 20 to 34 years old and who were singleton pregnant and first pregnancy. Blood samples were taken from patients once they were diagnosed with preeclampsia.
Recruitment	This study is based on the birth cohort in Shenzhen, China. The cohort was collected to assess the long-term cardiovascular risk of mothers and offspring exposed during pregnancy. Women with singleton pregnancies were recruited at 6 to 8 weeks of gestational age in Shenzhen Maternity and Child Health Care Hospital. Regular antenatal examination was performed.
Ethics oversight	This study was approved by the Ethics Committee of Shenzhen Maternity and Child Health Care Hospital (Shenzhen Maternal and Child Ethics Review SFYLS [2021]031).

Note that full information on the approval of the study protocol must also be provided in the manuscript.

Field-specific reporting

Please select the one below that is the best fit for your research. If you are not sure, read the appropriate sections before making your selection.

☒ Life sciences ☐ Behavioural & social sciences ☐ Ecological, evolutionary & environmental sciences

For a reference copy of the document with all sections, see nature.com/documents/nr-reporting-summary-flat.pdf

Life sciences study design

All studies must disclose on these points even when the disclosure is negative.

Sample size	23 pregnant women were subjected to scRNA-seq, including 8 patients with preeclampsia and 15 normal pregnant women. Since this is an exploratory study, sample size cannot be pre-determined precisely based on statistical methods.
Data exclusions	The genes expressed in more than 3 cells, and the cells with the number of genes detected ranged from 200 to 6000, with less than 10% of the mitochondrial genome and less than 1% of 10 hemoglobin genes (HBA1, HBA2, HBB, HBD, HBE1, HBG1, HBG2, HBM, HBQ1, HBZ) were retained.
Replication	This study lacks a description of replication as the experimental portion solely focuses on the preparation of single-cell suspensions and construction of sequencing libraries, which does not yield direct experimental outcomes. But all data analyses are reproducible.
Randomization	As this is a case/control study, randomization was not applicable for group allocation.
Blinding	Investigators who isolated PBMCs and constructed sequencing libraries for individuals were blinded to group allocation, but blinding was not applicable for investigators who performed comparison analysis between groups.

Reporting for specific materials, systems and methods

We require information from authors about some types of materials, experimental systems and methods used in many studies. Here, indicate whether each material, system or method listed is relevant to your study. If you are not sure if a list item applies to your research, read the appropriate section before selecting a response.

Materials & experimental systems

n/a	Involved in the study
☒	☐ Antibodies
☒	☐ Eukaryotic cell lines
☒	☐ Palaeontology and archaeology
☒	☐ Animals and other organisms
☒	☐ Clinical data
☒	☐ Dual use research of concern
☒	☐ Plants

Methods

n/a	Involved in the study
☒	☐ ChIP-seq
☒	☐ Flow cytometry
☒	☐ MRI-based neuroimaging

Plants

Seed stocks

Report on the source of all seed stocks or other plant material used. If applicable, state the seed stock centre and catalogue number. If plant specimens were collected from the field, describe the collection location, date and sampling procedures.

Novel plant genotypes

Describe the methods by which all novel plant genotypes were produced. This includes those generated by transgenic approaches, gene editing, chemical/radiation-based mutagenesis and hybridization. For transgenic lines, describe the transformation method, the number of independent lines analyzed and the generation upon which experiments were performed. For gene-edited lines, describe the editor used, the endogenous sequence targeted for editing, the targeting guide RNA sequence (if applicable) and how the editor was applied.

Authentication

Describe any authentication procedures for each seed stock used or novel genotype generated. Describe any experiments used to assess the effect of a mutation and, where applicable, how potential secondary effects (e.g. second site T-DNA insertions, mosiacism, off-target gene editing) were examined.
